# Hyperalgesia affects muscle activity and knee range of motion during a single-limb mini squat in individuals with knee osteoarthritis: a cross-sectional study

**DOI:** 10.1186/s12891-021-03947-w

**Published:** 2021-01-08

**Authors:** Jéssica Garcia Jorge, Ana Luiza Costa e Silva Cabral, Vanessa Martins Pereira Silva Moreira, Wallisen Tadashi Hattori, Valdeci Carlos Dionisio

**Affiliations:** 1 Laboratory of Neuromechanics and Physical Therapy, Faculty of Physical Education and Physiotherapy, Federal University of Uberlândia, Uberlândia, Brazil; 2 Medicine School at the Federal University of Uberlândia, Uberlândia, Brazil

**Keywords:** Hyperalgesia, Muscle activity, Postural control, Functionality, Knee osteoarthritis

## Abstract

**Background:**

The effect of hyperalgesia on functionality remains uncertain for individuals with knee osteoarthritis (KOA). This study aimed examine the clinical measures and hyperalgesia’s effect on muscle activity, knee range of motion (ROM) and postural control during the single-leg mini squat (SLMS) in individuals with KOA, determining the correlation between variables.

**Methods:**

In this cross-sectional study, 60 individuals, 30 healthy (HG, 57.4 ± 6.86 years), and 30 with mild to moderate KOA (KOAG, 59.4 ± 5.46 years) were evaluated by the visual analog scale (VAS), Western Ontario and McMaster Universities Index (WOMAC), and the pressure pain threshold (PPT) in subcutaneous, myotomal, and sclerotomal structures. Muscle activity, knee ROM and postural control were assessed during a SLMS. The analyses were performed in the two phases of the SLMS. Phase 1 - during descending movement (eccentric contraction), Phase 2 - during ascending movement (concentric contraction). Analysis of covariance was applied for each variable separately, using weight as a co-variable. We used Spearman’s test for determining the correlation.

**Results:**

There was no difference between groups for age, height, and postural control (*p* > 0.059), but KOAG presented the highest values for VAS and WOMAC (*p* = 0.000). In addition, EMG activity was higher in KOAG for gastrocnemius medialis and tibialis anterior muscles during phase 1 (*p* < 0.027), and for gastrocnemius medialis and gluteus medius muscles during phase 2 (*p* < 0.007), and reduced values for PPT and knee ROM (*p* = 0.000). Also, the correlations between PPT with muscle activity and postural control were moderate (rho< 0.482), while strong relationships were observed between some PPT points with VAS and WOMAC (rho> 0.507).

**Conclusion:**

Hyperalgesia affects the functionality during a single-limb mini squat. There is an important correlation between hyperalgesia and muscle activity, postural control, and clinical measures in individuals with KOA.

## Background

Knee osteoarthritis (KOA) tends to increase considerably worldwide due to aging and obesity [1]. Pain is the main symptom of KOA, which is associated initially with local peripheral sensitization, but also can become chronic, promoting a neurological reorganization within the segments of the spinal cord and the cortical level [2–4]. The central nervous system can then become hypersensitive to non-nociceptive stimuli in a process called central sensitization characterized that affects distant areas of the knee [5–7]. Thus, increased peripheral and central pain are classified as hyperalgesia [6].

Each segment of the hypersensitized spinal cord and its corresponding spinal nerves have a segmental relationship, which allows determining the likely level of spinal dysfunction based on the pattern of dermatomal, myotomal, or sclerotomal hyperalgesia [7, 8]. Although hyperalgesia could be assessed by the pressure pain threshold (PPT) [9], this segmental relationship has been poorly explored [8]. Understanding it can be considered the best way to recognize the sensory abnormalities [7] since pain is one of the main causes of functional limitation and disability in KOA [1].

Functional limitations in KOA include restrictions on walking [10], ascending/descending stairs [11, 12], bipedal squat [13, 14] and any other weight-bearing activity on the affected limb [14], such as single-limb mini squat (SLMS). Weight-bearing asymmetry [13], muscle weakness [11, 12], adaptative trunk position [15] and poor proprioception [11] have been observed in individuals with KOA. The SLMS is a more challenging task whereas is not possible to redistribute body weight and can reveal electromyographic (EMG), kinematic and kinetic differences [16, 17]. The SLMS is used in knee rehabilitation programs [16], in assessments of movement quality, dynamic alignment, postural control and stability [16, 18]. Despite this, it has been little evaluated in individuals with KOA.

Evaluating postural control in challenging positions, such as weight-bearing in a single limb, has been shown to predict decreased functionality in individuals with KOA [18]. In this way, pain could change postural control by reducing the muscle’s ability to maintain stability, altering muscle recruitment and activation [10, 19]. The theory of spiraling pain, characterized by maintaining a sustained contraction caused by the disinhibition and sensitization of gamma motoneurons leading to ischemia and more pain [19], could explain this muscular alteration. This disinhibition could also be related to a cognitive and behavioral influence in a fear-avoidance model [20]. However, although these theories have been proposed, few studies have evaluated the relationship between pain and EMG activity [21] and pain and postural control [19, 22] in individuals with KOA. In all of these previous studies, pain was experimentally induced or assessed through clinical measures (questionnaires and/or scales). Thus, up to date, no study has been found that analyzed the segmental relationship of hyperalgesia through PPT with muscle activity, postural control and other clinical measures. This relationship is essential, since it involves knee flexion and weight-bearing movements, with pain exacerbation in KOA, commonly reported [23]. The clarification of these relationships could be necessary to decrease the pain and improve individuals’ functionality with KOA during rehabilitation exercises and daily life activities, directing a more specific clinical intervention.

Here we hypothesized that 1) hyperalgesia would increase muscle activity, but the knee flexion displacement and postural control would reduce during the SLMS in individuals with KOA; 2) clinical measures and hyperalgesia would be related muscle activity and postural control. Thus, this study aimed examine the clinical measures and hyperalgesia’s effect on muscle activity, knee range of motion (ROM) and postural control during the single-leg mini squat (SLMS) in individuals with KOA, determining the correlation between variables.

## Methods

### Design and participants

In this cross-sectional study, 60 individuals (Table 1) signed an informed consent for participation in this study, approved by the Human Research Ethics Committee at the Federal University of Uberlandia (CAAE 37398414.6.0000.5152). Thirty participants were allocated for the healthy group (HG) and 30 for the KOA group (KOAG). The eligibility criteria for KOAG were a range from 50 to 70 years; KOA (unilateral or bilateral) at a mild or moderate level, according to the requirements of the American College of Rheumatology [24]; knee pain for 6 months or more [25]; and a minimum score of 4 points on visual analog scale (VAS) [2]. The individuals would not be presented with other musculoskeletal disorders, diabetes mellitus, neurological/mental disorders, or using drugs which side effects affect the sensory capacity and control movement abilities. The participants could not have performed physical therapy intervention or physical activity for at least 3 months. Also, they could not use analgesics in the last 4 h before data collection [4]. For HG, the participants should be in the same age range but without diabetes mellitus, musculoskeletal (including KOA), or neurological/mental disorders.

**Table 1:** Characteristics of the participants

Variables	HG (***n*** = 30) Mean ± SD	KOAG (***n*** = 30) Mean ± SD	***F***	***p-value***
Age (years)	57.4 ± 6.86	59.4 ± 5.46	1.608	0.210
Height (m)	1.63 ± 0.10	1.66 ± 0.08	0.987	0.325
Weight (Kg)	68.87 ± 10.89	78.93 ± 14.99	8.534	0.005*
Body mass index	25.64 ± 3.36	28.40 ± 4.30	7.669	0.008*
VAS	0.36 ± 0.91	5.93 ± 1.69	243,242	0.000*
WOMAC	1.66 ± 4.43	33.54 ± 21.20	62.799	0.000*
Sex	Men (15)	Men (15)		
	Women (15)	Women (15)		
Involvement	–	Bilateral (14):		
		11(R) 3 (L)		
		Unilateral (16):		
		10 (R) 6 (L)		

*HG* Healthy Group, *KOAG* Knee osteoarthritis group, *VAS* visual analog scale, *WOMAC* Western Ontario and McMaster Universities Index, *R* right, *L* left; *p-value≤0.05

### Evaluation of pressure pain threshold (PPT)

A pressure algometer (EMG System Brazil Ltda®) with a flat head of ½ inch in diameter, 20 kg capacity and was used to evaluate the PPT according to the previous studies [5, 7]. The dermatomal, myotomes, and sclerotomal points followed the previous protocol [2]. For dermatomal hyperalgesia, PPTs were measured at the third (L3) and fourth (L4) lumbar vertebrae levels and second vertebra sacral (S2). The myotomes were selected according to their anatomical location, referent the knee (close and distant). The rectus femoris and tibialis anterior were located close to the knee, above and below. The adductor longus and quadratus lumborum muscles were considered as a distant location to the knee. Finally, the sclerotomal hyperalgesia was evaluated in the pes anserinus bursae and patellar tendon. A digital metronome with 1 Hz was used in all evaluations to standardize the application rate of pressure. The PPT was expressed in kgf/cm2, and the highest PPT values indicated less severe symptoms.

### Electromyography and kinematics

Active superficial bipolar electrodes and pre-amplified were used with a gain of 20 times, armored cable, and pressure clip at the end. Their placement was performed according to the SENIAM - Surface Electromyography for the Non-Invasive Assessment of Muscles - BIOMED II [26] proposal in muscles of the affected lower limb or with a more substantial complaint of pain in KOA [21]. For healthy participants, the dominant lower limb was assessed. The EMG activity of the gastrocnemius medialis, biceps femoris, erector spinae (longissimus), gluteus medius, rectus femoris, vastus medialis, vastus lateralis, and tibialis anterior muscles was registered at a sampling frequency of 2 kHz and stored in the computer. The signals were captured by a signal acquisition system (EMG System Brazil Ltda®) of 12 channels conditioned with analog filters (Butterworth - 4th order) with a cut-frequency band (20–500 Hz) and signal input noise level < 3 μV RMS. The equipment had a 100 times amplifier gain, and the total amplification gain was 2000 times. The input impedance was 109 Ohms, and a common-mode rejection ratio > 100 dB.

**Fig. 1:**
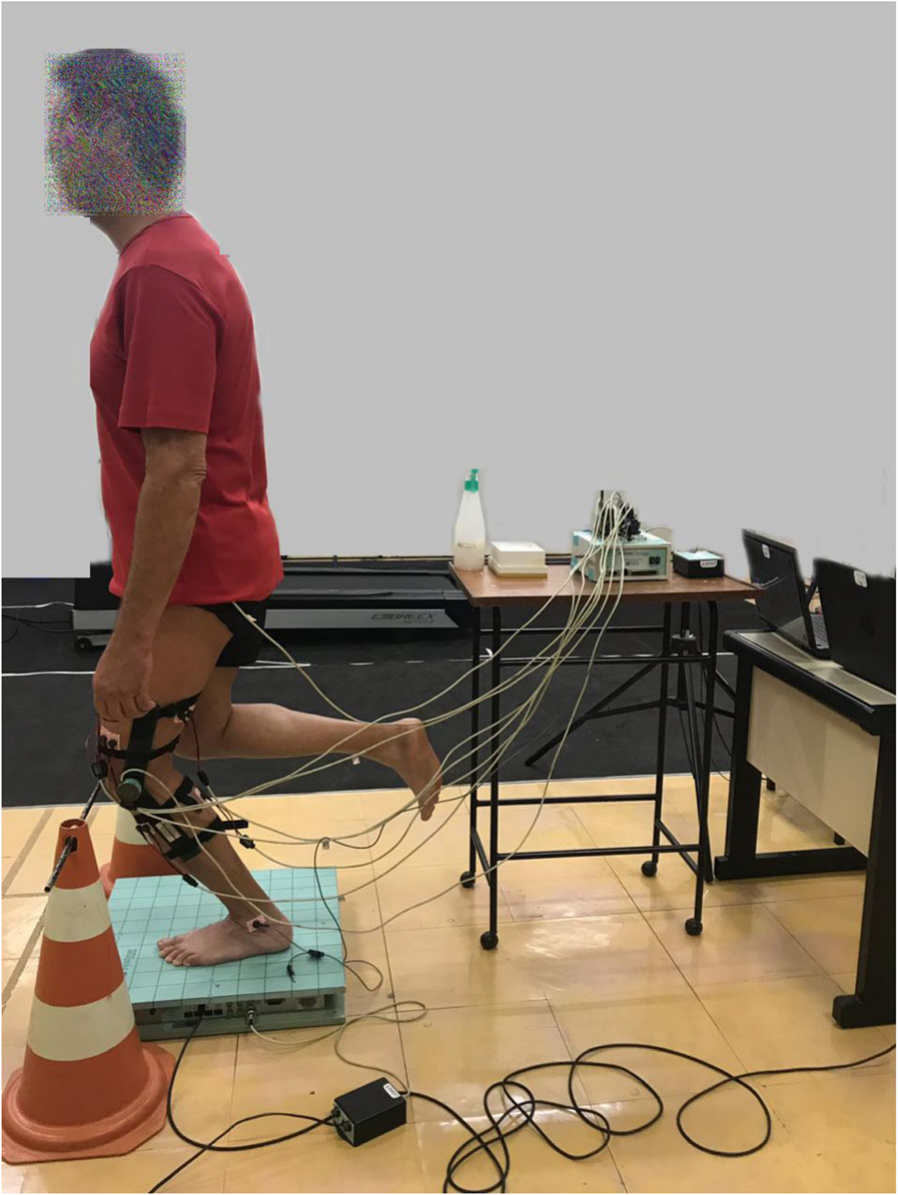
This experimental set shows the individual on top of a force platform with electrodes and an electrogoniometer attached, performing the single-leg mini squat

The acquisition system also received and synchronized the kinematic signals by an electrogoniometer (EMG System Brazil Ltda®) with flexible poles and rotation of 270° positioned in the lateral epicondyle of the femur, with the stems aligned to the greater trochanter of the femur and lateral malleolus. The positive value represented the knee flexion and the negative value knee extension (Fig. 1).

Kinematic and EMG data were treated in MatLab environment software 7.1 (The Math Works, Inc. Natick, MA, USA). The EMG data were rectified, filtered, normalized by the linear envelope, and peak activity of each muscle [27]. The normalized EMG activity was integrated based on knee displacement in two distinct phases. Phase 1 during descending movement (eccentric contraction) from beginning to the peak of the movement. Phase 2 during ascending movement (concentric contraction) from the peak to the end of the movement.

### Postural control

For the postural control evaluation, the force plate (BIOMEC 410 -EMG System Brazil software - version 2013) was used with a sampling frequency of 100 Hz. The analog signal sent through an amplifier was converted to a digital signal (24-bit A/D). The device’s accuracy was 0.5%, and its dimensions 500 × 500 mm, following the forward direction (Y+) and right side (X+) coordinates. The center of pressure displacement was processed by software (EMG System Brazil Ltda®) and filtered by a Butterworth filter at 10 Hz. The variables analyzed were the elliptic area (cm^2^), displacement (cm), amplitude (cm) and velocity (cm/s) in anteroposterior (AP), and mediolateral (ML) directions.

### Experimental procedure

Initially, the personal and anthropometric data were recorded, and then, the VAS and Western Ontario and McMaster Universities Index (WOMAC) questionnaire were applied (clinical measures). Subsequently, each participant was laid on a stretcher, and the PPT was measured. After familiarization with the stimulus by performing two practical tests on their forearm [7]. The measurements were conducted once and bilaterally [2, 25] and in a randomized order (Microsoft Excel 2013), in the dermatomes, myotomes, and sclerotomes. After that, the EMG electrodes and the electrogoniometer were positioned. The participants were placed over the force plate with bare feet and facing forward (Y), and arms extended along the body [22] (Fig. 1). After brief familiarization with the test, they were instructed to perform the SLMS with the affected or complained of pain limb in the KOAG or dominant limb in the HG. The movement’s instruction was “do it as fast as possible and how much you can”. The target was a maximal of 45° of knee flexion restricted by a flexible obstacle placed in front of the knee. The EMG, kinematic, and kinetic signals were recorded simultaneously after a verbal command: “Prepare ... go!” Three repetitions of 10-s were performed [21]. Between each record, a 30-s interval was permitted. The participant remained with open eyes during the task, fixing the gaze target at eye level three meters away [28].

### Statistical analysis

The prior calculation of the sample size indicated that at least 23 participants were required for each group. For calculation, we used the software G*Power 3.1.2.9 (Franz Faul, Universität Kiel, Germany), using the family F tests, ANOVA: fixed effects, one way, with a statistical power 0.90 at an effect size of 0.50 with an alpha level of 0.05. The normal distribution was tested and confirmed by the Shapiro-Wilk test. The analysis of variance one-way was applied for each variable separately (VAS, WOMAC, PPTs). Analysis of covariance (ANCOVA) was applied for postural and EMG data. Each muscle in each phase was tested separately, using the weight as a co-variable to avoid this confounding factor. For the correlation test, Spearman’s correlation coefficient was used because, in some situations, the model’s assumptions (normality, homogeneity, and independence of the residues) were not met. It was considered a weak correlation rho < 0.4, moderate correlation rho > 0.4 to < 0.69, and strong correlation rho > 0.7 [29]. All the tests were performed on IBM SPSS© (version 22.0.0.0) with a significance level of 0.05.

## Results

The results showed that there were no differences between the groups regarding age and height. However, the KOAG had higher weight, higher WOMAC and VAS scores (Table 1), and lower PPTs values than the HG (Table 2).

**Table 2:** Pressure pain threshold values

PPTs	HG Mean ± SD	KOAG Mean ± SD	***F***	***p-value***
Dermatomes (kg/cm2)				
L3_R	2.803 ± 1.638	1.675 ± 1.672	6.953	0.011*
L3_L	2.862 ± 1.369	2.105 ± 2.316	2.370	0.129
L4_R	1.814 ± 1.074	0.758 ± 0.549	22.947	0.000*
L4_L	1.972 ± 0.897	0.674 ± 0.406	52.040	0.000*
S2_R	3.247 ± 1.574	2.047 ± 1.170	11.221	0.001*
S2_L	3.256 ± 1.481	2.204 ± 1.283	8.638	0.005*
Myotomes (kg/cm2)				
Rectus femoris _R	5.747 ± 2.749	3.738 ± 2.223	9.681	0.003*
Rectus femoris_L	5.654 ± 2.309	3.938 ± 1.935	9.731	0.003*
Tibialis anterior_R	4.905 ± 2.395	3.279 ± 1.654	9.360	0.003*
Tibialis anterior_L	5.174 ± 2.711	4.033 ± 1.685	3.836	0.050*
Adductor longus_R	3.116 ± 1.805	1.794 ± 1.429	9.877	0.003*
Adductor longus_L	3.482 ± 1.883	2.191 ± 1.739	7.605	0.002*
Quadratus lumborum_R	5.049 ± 2.204	4.001 ± 2.075	3.598	0.063
Quadratus lumborum_L	5.271 ± 2.544	4.410 ± 2.432	1.795	0.186
Sclerotomes (kg/cm2)				
P*es anserinus* bursae_R	3.448 ± 1.802	1.890 ± 0.974	17.364	0.000*
P*es anserinus* bursae_L	3.806 ± 1.902	2.893 ± 1.669	3.907	0.050*
Patellar tendon_R	7.303 ± 3.478	3.976 ± 2.319	19.002	0.000*
Patellar tendon_L	7.306 ± 2.770	4.999 ± 2.781	10.853	0.002*

*HG* Healthy group, *KOAG* Knee osteoarthritis group, *L3* third lumbar level, *L4* fourth lumbar level, *S2* second sacral level, *R* right, *L* left; *p-value≤0.05

**Fig. 2:**
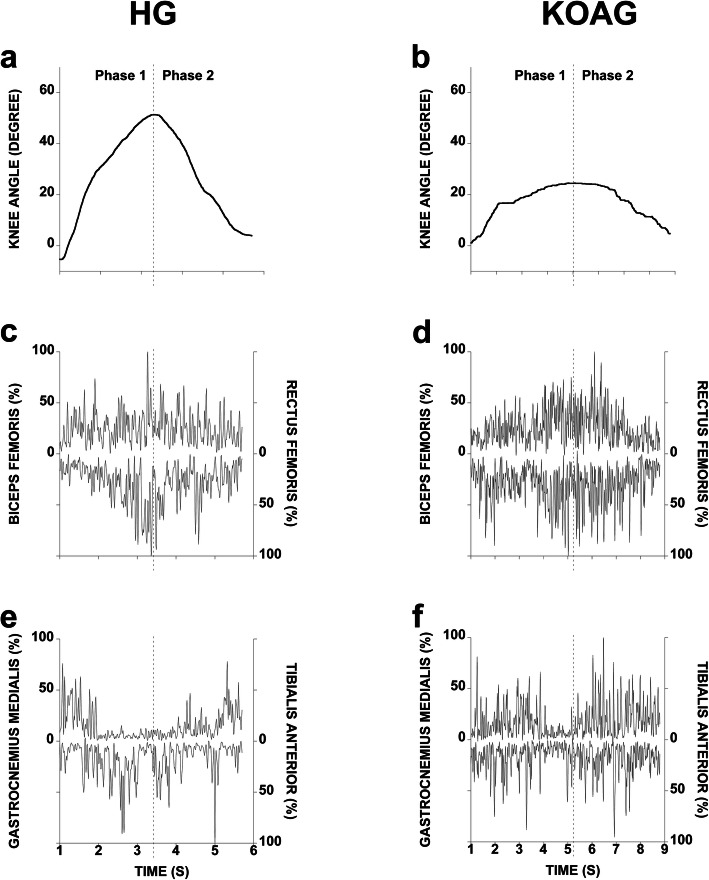
This picture describes the knee angular displacement (**a** and **b**); biceps femoris and rectus femoris (**c** and **d**), tibialis anterior and gastrocnemius medialis (**e** and **f**) muscles activities during a single-limb mini squat, which was performed by one participant in each group. The vertical dotted line delimits the squat phases. HG: healthy group; KOAG: knee osteoarthritis group

The comparison between groups also revealed that during the SLMS, the KOAG (32.28 ± 7.47) presented lower knee ROM (t = 5.740; *p* = 0.000) compared to the HG (42.90 ± 6.83) and spent more time to perform the task (Fig. 2a and b).

On the other hand, the integrated EMG (iEMG) activity was higher in KOAG. There was an increase in the iEMG for gastrocnemius medialis and tibialis anterior muscles during phase 1 and gastrocnemius medialis and gluteus medius muscles during phase 2 (Table 3).

**Table 3:** Integrated muscle electromyography activity during the single-limb mini squat

Muscles	HG Mean ± SD	KOAG Mean ± SD	***F***	***p-value***
Phase 1 (eccentric contraction)				
Gastrocnemius medialis	0.136 ± 0.062	0.176 ± 0.064	6.526	0.013*
Erector spinae_R	0.194 ± 0.072	0.210 ± 0.069	0.872	0.354
Erector spinae_L	0.191 ± 0.048	0.218 ± 0.055	3.799	0.056
Gluteus medius	0.166 ± 0.062	0.163 ± 0.052	0.003	0.956
Rectus femoris	0.238 ± 0.069	0.273 ± 0.060	1.791	0.186
Vastus medialis	0.282 ± 0.053	0.278 ± 0.064	0.388	0.536
Vastus lateralis	0.925 ± 0.079	0.892 ± 0.130	1.160	0.286
Tibialis anterior	0.118 ± 0.068	0.165 ± 0.074	5.167	0.027*
Biceps femoris	0.202 ± 0.070	0.181 ± 0.079	0.239	0.627
Phase 2 (concentric contraction)				
Gastrocnemius medialis	0.070 ± 0.051	0.116 ± 0.059	7.979	0.007*
Erector spinae _R	0.135 ± 0.054	0.136 ± 0.057	0.581	0.449
Erector spinae _L	0.256 ± 0.054	0.262 ± 0.045	0.418	0.521
Gluteus medius	0.130 ± 0.093	0.228 ± 0.113	9.999	0.003*
Rectus femoris	0.294 ± 0.052	0.302 ± 0.051	0.609	0.438
Vastus medialis	0.171 ± 0.050	0.184 ± 0.053	0.932	0.338
Vastus lateralis	0.218 ± 0.094	0.241 ± 0.067	0.527	0.471
Tibialis anterior	0.183 ± 0.077	0.209 ± 0.076	1.820	0.183
Biceps femoris	0.111 ± 0.061	0.125 ± 0.072	1.706	0.197

*HG* Healthy group, *KOAG* Knee osteoarthritis group, *R* right, *L* left. *p-value≤0.05. Covariates appearing in the model are evaluated at the following values: Weight = 73.90

**Fig. 3:**
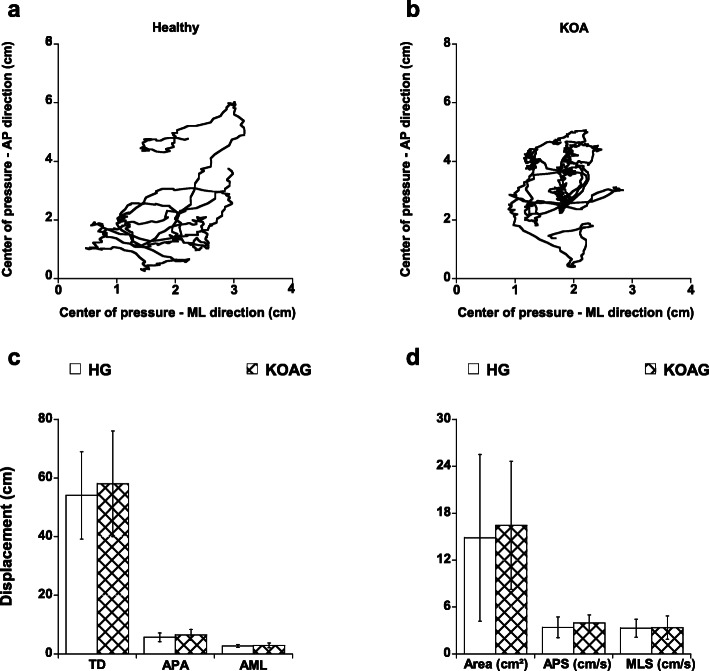
This picture describes the center of pressure displacement in anteroposterior and medio-lateral directions for one individual of the healthy (**a**) and one with knee osteoarthritis (**b**) group. Also, we are showing the mean and standard deviation across all participants for healthy and knee osteoarthritis groups of the TD - total displacement; APA – amplitude in anteroposterior direction; AML – amplitude in medio-lateral direction (**c**), and the area, APS – anteroposterior speed and MLS – medio-lateral speed (**d**)

For postural control, there were no statistical differences (*p* > 0.059) between the groups for all variables analyzed (Fig. 3).

The results also showed that there was a moderate correlation between hyperalgesia and iEMG activity (rho < 0.482) and hyperalgesia and postural control (rho < − 0.436). However, there were a moderate to strong correlations between PPT values in dermatome L4 (VAS, rho = 0.695; WOMAC, rho = 0.701), adductor longus myotome (VAS, rho = 0.557; WOMAC, rho = 0.594) and patellar tendon sclerotome (VAS, rho = 0.565; WOMAC, rho = 0.507).

## Discussion

This study aimed examine clinical measures and hyperalgesia’s effect on muscle activity, knee range of motion (ROM) and postural control during the SLMS in individuals with KOA, determining the correlation between variables. Our results showed that the KOAG presented the highest VAS and WOMAC scores and the decrease of PPTs. Also, KOAG presented increased iEMG activity in some muscles and reduced knee ROM during SLMS. The correlations between PPT with muscle activity and postural control were moderate. However, strong relationships were observed between some PPTs points, VAS, and WOMAC.

In the comparison between groups, the highest scores of VAS and WOMAC (Table 1) and the decrease of PPTs (Table 2) in close and distant points from the knee, in both limbs, indicated the presence of hyperalgesia in individuals with KOA. These results corroborate with several other findings [2, 7, 22, 30]. The hyperalgesia found in the KOAG could have been triggered, not only by the reorganization of the nociceptive pathways within the spinal cord segments [8], but also by the influence of cognitive factors and emotional (mood, stress, anxiety, and depression alterations) capable of altering the perception of pain [3, 31]. These factors, although not evaluated in the present study, may modify activation patterns in cerebral cortical regions, especially in the limbic and prefrontal areas that influence the somatosensory cortex [6, 32–34].

The cortical motor centers can also be further stimulated by the same process when under conditions of psychic stress and fear. Stimulating gamma motoneurons by descending pathways to increase tone and muscle activity within a vicious cycle involving pain and sustained co-contraction [6, 21, 34]. These mechanisms could explain the higher EMG activity in KOAG when compared to HG (Table 3). The pattern of sustained contraction and muscle coactivation suggested in our findings (Fig. 1; Table 3) are similar to previous studies [4, 17, 35]. The stress during the task execution, associated with the probable coactivation pattern aiming to stabilize the limb [14], was probably caused by the complexity of the squat task [36], added to the fear of pain [37] and the attempt to maintain postural control [11].

This attempt to stabilize the knee due to greater muscle activation and coactivation during weight acceptance, suggested in this study, is consistent with other studies [4, 17, 36, 38] and occurs mainly during small ROM [39, 40]. Our results showed that the KOAG presented higher muscle activation than HG (Table 3), even though the squatting was performed slower and in a smaller knee ROM (Fig. 1). The muscle activation and coactivation could be generalized motor control strategy, and less related ROM and velocity [41] during knee stabilization increasing joint stiffness [39, 40]. This strategy was intensified by delayed muscle activation and deactivation when compared to healthy individuals [42]. Thus, the differences between groups concerning smaller angular displacement could also be justified by fear and higher muscle activation.

Our results showed moderate correlations between hyperalgesia with muscle activity and postural control, suggesting that the hyperalgesia is not the main factor of changes in these variables in individuals with KOA [22, 28]. Those variables may relate more to the peripheral alterations due to the disease severity [23, 43], and the cortical modifications due to the influence of cognitive and emotional factors [3, 20], which are independent of the presence of the painful stimulus. This study is the first to trace such correlation, which is relevant to direct a more specific clinical approach of individuals with the disease. For example, hyperalgesia is not the main cause of changes in the variables studied. The clinicians should pay attention to other influencing factors, like the peripheral changes related to acute pain and cognitive and emotional factors. Peripherical changes as the inflammatory process and joint wear would exacerbate pain during challenging movements (such as SLMS) [22, 23, 28, 44]. Also, the influence of cognition, emotion, and fear of movement stimulate cortical regions and the descending pathways of motor control, changing the muscular response [3, 34]. Guiding the clinical approach to these two factors could produce better treatment results.

Our results showed a moderate to a strong correlation between L4, adductor longus, patellar tendon points, and clinical measures. These points could indicate pain and functionality (VAS and WOMAC), corroborating partially with the previous study’s findings [2]. These results would be important, as it would enable clinicians to select these points for pain assessment of central origin previously. However, further studies are needed to test this hypothesis.

The lack of trunk kinematic analysis could be considered a study limitation since the AP displacement influences muscle activity, especially the biceps femoris [35, 40]. However, the EMG activity of biceps femoris was similar between groups, suggesting the trunk was stable during the study’s tests. Future studies analyzing the kinematics of trunk and lower limbs in different movement plans would provide a big picture of SLMS in individuals with KOA.

This study provides important information on hyperalgesia, biomechanical factors, and clinical measures. The focus on pain, joint mobilization, muscle strengthening, and neuromuscular training seem appropriate for the physiotherapeutic approach. However, this result also reinforces the idea that cognitive and emotional aspects can influence clinical measures. Thus, clinicians should take these aspects into account to achieve better management of individuals with KOA.

## Conclusion

Hyperalgesia affects the functionality during a single-limb mini squat. There is an important correlation between hyperalgesia and muscle activity, postural control, and clinical measures in individuals with KOA.

## Data Availability

The datasets used and/or analyzed during the current study are available from the corresponding author on reasonable request.

## Abbreviations

EMG: Electromyographic
HG: Healthy group
KOA: Knee osteoarthritis
KOAG: KOA group
L: Left
L3: Third lumbar vertebrae level
L4: Fourth lumbar vertebrae level
ML: Medio-lateral
PPT: Pressure pain threshold
R: Right
ROM: Range of motion
S2: Second vertebra sacral level
SLMS: Single-limb mini squat
VAS: Visual analog scale
WOMAC: Western Ontario and McMaster Universities Index
